# Outcomes of brace treatment for adolescent idiopathic scoliosis with curve magnitude of 41 to 50 degrees

**DOI:** 10.1186/1748-7161-9-S1-O22

**Published:** 2014-12-04

**Authors:** Toru Maruyama, Yosuke Kobayashi, Makoto Miura, Yuske Nakao

**Affiliations:** 1 Saitama Medical University, Kawagoe, Japan

## Background

Effectiveness of brace treatment for adolescent idiopathic scoliosis (AIS) patients with curve magnitude of 20 to 40 degrees was demonstrated by BRAIST study in 2013. However, its effectiveness for the curve over 40 degrees was under controversy.

## Aim

To investigate outcomes of brace treatment for AIS patients with curve magnitude of 41 to 50 degrees.

## Design

Case series from prospectively constructed data base.

## Methods

AIS patients with age over 10 years, Risser sign of 0 to II, within one year postmenarche, Cobb angle of 41 to 50 degrees before treatment and underwent no prior treatment were included in the study. At the final follow-up after the patients reached skeletal maturity, the rate of the patients whose curve was stabilized by the treatment (the curve had not progressed in more than 6 degrees), whose curve exceeded 45 degrees and who underwent surgery were investigated.

## Results

A total of 12 female patients was included in the analysis. The average age was 12.3 years (11-15) and the average Cobb angle was 45.0 degrees (41 to 50) before treatment. Risser sign was 0 in three, I in five, and II in four patients. There were eight thoracic, two thoracolumbar, and two double major curves. Initial correction rate by the brace was 35.5%. After an average follow-up period of 36 months, the average Cobb angle changed to 50.3 degrees. The curve of six patients (50%) was stabilized by the treatment. The curve of nine patients (75%) exceeded 45 degrees and three patients (25%) underwent surgery.

## Discussion

For the curve magnitude of 25 to 40 degrees, the rate of the patients whose curve was stabilized by the brace treatment was 79% in our institution. Comparing with these results, the stabilization rate (50%) of the present study for the curve of 41 to 50 degrees was relatively low. However, the progression rate of such magnitude curve in the natural history was reported to be 70-90%.

## Conclusions

Fifty per cent of the curves of 41 to 50 degrees was stabilized by the brace treatment, which had some effectiveness even for the curve of such magnitude.

